# Families’ Experiences on Safety Needs of Children with Intellectual Disability

**DOI:** 10.3390/ijerph192215246

**Published:** 2022-11-18

**Authors:** Mantji Juliah Modula, Gsakani Olivia Sumbane

**Affiliations:** 1 School of Nursing, Faculty of Health Sciences, University of Free State, Bloemfontein 9300, South Africa; 2 School of Medicine, Faculty of Health Sciences, University of Limpopo, Polokwane 0727, South Africa

**Keywords:** children, safe environment, families, intellectual disability, need, safety, protection, security

## Abstract

Background: Children with intellectual disability (ID) are known to have a deficit in self-care, social interaction, and learning abilities. Families raising these children experience a range of difficulties that require supportive systems to meet the physical, psychological, and social safety rights of children with ID. The study explored the safety of children with ID through the experiences of their families in the rural Capricorn District of Limpopo Province, South Africa. Methods: In-depth individual interviews and focus group discussion were conducted with 26 families directly involved in raising the children with ID. An inductive thematic analysis of data on the experiences of raising children with ID was undertaken with the aid of ATLAS.ti 8 computer programme. Results: The study revealed that children with ID lack safety at home, schools, and day care centre environments due to a lack of active involvement by nuclear family members, neighbours, and communities, including interaction with their peers and professional service providers in facilities. Safety of children with ID is compromised through exploitation and injuries, leading to marginalisation as they feared further humiliation. Conclusion: The study highlighted that active involvement of family members, communities, and governmental and non-governmental organisations is crucial in ensuring safe environments for children with ID.

## 1. Introduction

Intellectual disability (ID) is emblematic of a deficit or decrease in neurodevelopmental functioning and is distinguishable by its significant intellectual and adaptive behavioural limitations that emerge before the age of 18 [1]. The intellectual functioning assessment of ID focuses on cognitive abilities, including problem solving and reasoning acumen; adaptive behaviour includes conceptual, practical, and social domains such as self-care, social interaction, and learning abilities [2].

Factors such as immature judgement and abstract thinking are a demonstration of a deficiency in the interpretation of the environment and an individual’s inability to self-protect [1,3]. The social domain deficit in the child with ID expresses itself through communication, linguistic, and conversational difficulties, which result in a difficulty to regulate one’s own emotions and behaviour. Furthermore, poor social judgment in these children limits their understanding of risks in social environments and exposes them to manipulation by others (gullibility) as they struggle to express their rights [4], compromising their safety. The deficit in ID-related cognitive functioning is a causative factor of reduced knowledge about personal safety skills, which subjects these children to maltreatment by others [5]. Nonetheless, disability is not only confined to the bodily impairment of these children, but the political and social environments are also linked to the exclusion of the children with ID’s involvement and participation in societal activities and programmes crucial for human development [6].

The concept of safety is located primarily on protection and prevention of physical and psychological harm within one’s environment, as well as ensuring comfort and freedom from fear [7]. Furthermore, Maslow’s theory of hierarchical needs posits safety and security needs as the most basic human need whose fulfilment engenders satisfaction of the human being’s physiological needs [3]. This theory identified the safety and security needs as those of avoiding harm, maintaining comfort, physical safety, order structure, protection, and freedom from fear. Furthermore, failure to fulfil these hierarchically ordered needs eventually disrupts one’s progress towards other levels of love, belonging, self-esteem, and self-actualisation [3]. Studies found that children with ID have low safety abilities resulting from difficulty in learning safety skills required for self-defence [8]. In addition, the safety of the children with ID focused largely on their physical and relational well-being, as well as receiving assistance from people they trust to eliminate the risk posed by both strangers and those they know [9].

The United Nations Convention on the Rights of Persons with Disability (UNCRPD) further promotes the safety rights of children with ID by ensuring respect for their human dignity and protection from abuse and exploitation, including forced labour [10]. These rights are enforced by United Nations Conventions on the Rights of Children (UNCRC), which enjoin all member states to protect and provide all children with ID with proper care through development and implementation of legislative measures that prohibit maltreatment against them [11].

In South Africa, the increase in ID’s prevalence is not clearly indicated due to the lack of accurate epidemiological data [12,13]. Notwithstanding, the country is implementing various regulatory mechanisms and institutional/organisational involvement to ensure protection, safety, and security of persons with intellectual and physical disabilities. The Department of Social Development (DoSD)/Department of Women, Children and People with Disabilities (DCWPD)/UNICEF (2012) assert that people living with disability are likely to be the victims of rape and sexual assault, and some reported court cases were not thoroughly investigated owing to their vulnerability as witnesses [14]. The South African Constitution (1996) and the White Paper on Persons with Disabilities (2016) further advocate for the rights of children with ID and their protection against abuse and exploitation from their families, institutions caring for them (including schools and health care services), and any member of society [15]. Thus, promotion of these rights confirms and underscores the obligation to protect the children with ID from unjust treatment and further encourages access to health, rehabilitation, and education services [11].

Due to the decision-making deficit among children with ID, their survival needs depend mostly on their family members as primary caregivers in a home environment. However, families raising children with ID require support from communities to function effectively as a unit [16]. Studies have established that the existence of a child with ID in families highlights the beginning of a journey that requires adjustments by the whole family [17]. Previous studies indicate that the child with ID enters the family and creates inevitable crisis of the family system, resulting in a sense of tragedy that dismantles its stabilised functioning level [18]. In most cases, a home environment for upbringing of children in African families requires the involvement of the community, including family members, extended families, neighbours, friends, and in-laws [19]. The study by Briggs and Hawkins (2005, as cited in Robinson and Graham, 2021) [9], also found that children with ID were at risk of violence in their own homes and schools and from peers and their safety is a struggle for their families. As such, the increasing prevalence of people with ID globally motivated the researchers to conduct this study to explore and describe the safety of children with ID in rural areas with limited resources, such as the Capricorn District of Limpopo Province, South Africa.

Studies have shown that children living with ID continue to experience social stigma and discrimination, which compromise their basic human right to safety and security [13], compared to their peers without ID. Currently, there are few studies on the safety of children with ID. Hence, exploring their safety needs and experiences will contribute to the body of information and knowledge with respect to reducing their neglect, exploitation, and abuse. It is noteworthy that the vulnerability of children with ID subjects them to avoidable and preventable harm compared to their peers without ID [20], significantly exposing them to a higher risk of being the victims of multiple incidents of neglect, trauma, and abuse [8]. Furthermore, social stigma and cultural beliefs towards people with disabilities have impacted on the community support provided to the families who raise children with ID [21]. Regrettably, there is limited literature focusing on the experiences of the safety of children living with ID [9], which is fundamentally the motivation for the current qualitative study undertaken to explore the safety of children with ID through the experiences of their families raising them in the rural areas of Capricorn District of Limpopo Province, South Africa.

## 2. Materials and Methods

### 2.1. Research Design

A qualitative research design was adopted to explore and describe the experiences of families regarding the safety needs of their children with ID. A semi-structured interview guide was utilised during in-depth individual face-to-face interviews and focus group discussion with the sampled families directly raising and caring for children with ID in the home environment [22]. The approach was suitable to provide information on these families’ views regarding the safety of their children with ID.

### 2.2. Participant Recruitment

Twenty-six participants were recruited from a sample of families raising children with ID in the rural villages of Capricorn District, Limpopo Province. Participants were recruited from multiple sites consisting of villages under tribal authorities and municipalities in Capricorn District. The district is the economic hub of Limpopo Province, and offers better education, delivery of health care and other services, as well as work opportunities compared to other districts in Limpopo Province. The core function and common denominator among these families as caregivers are that they were raising and providing direct care to children with ID. Participants were recruited primarily through mental health clinics and day care centres’ attendance registers. The snowball sampling method was utilised to reach and recruit those potential families raising children with ID not known in the community facilities.

The eligibility criteria were premised on the inclusion of family members above 18 years of age, who were willing and consented to participate in the study and also lived with and provided direct care to the children with ID in the home environment. Furthermore, the researchers found it instrumental, according to their own judgement, to include the families with children over 6 years of age and a diagnosis of ID already confirmed either by health care providers, early childhood development centres, or schools. It was the opinion of the researchers that, at this age, their developmental abilities can be compared with their peers of school-going age. Such families were regarded by the researchers as having adequate experiences and information on raising children with ID. Eventually, the family members participating in this study consisted of 16 mothers, 1 father, 2 guardians, 3 aunts, 1 grandmother, 1 grandfather, and 2 uncles rearing children with ID. All participants were directly involved in raising children with ID.

Table 1 below indicates the socio-demographic information of participants showing that the care and safety of the children with ID were often the responsibilities of mothers who were not working, at 61.5%. Most children, at 69.2%, were staying at home and not attending any schools or day care centres. Mostly, members of these families caring for the children with ID were female primary caregivers, at 84.6%. These families were often single-parent families, at 42%, indicating a high rate of female-headed households.

### 2.3. Data Collection

The study conducted 16 individual in-depth interviews as the primary research method and focus group discussion of 10 participants as the follow up with the sampled members of families raising children with ID. The study explored their experiences on the safety needs of their children with ID. The focus group discussion consisted of female participants only and was as homogenous as possible to allow them freedom to share and express their opinions and experiences of raising their children with ID [22,23]. Factors such as gender, age, educational level, and relationship to the child with ID contributed to the selection of focus group discussion participants to promote spontaneity and interaction on their experiences [23]. The focus group interview further allowed collection of diversified information on the safety of children with ID from participants who had knowledge of raising children with ID [24]. The individual interviews were conducted in the participants’ natural home environments where the children are raised by these families. The focus group was held at the home of one of the participants who was willing to attend but could not leave her child alone. The natural environment allowed the researchers to understand the context in which the families raise their children with ID [25].

Family members who participated in individual interviews were excluded from the focus group discussion to allow comparability of data [22]. Data from individual interviews were used to form a baseline to conduct focus group discussion. Semi-structured interview guides were designed for both interviews and were tested through piloting and questions were refined into a final version with an assistant intellectual disability expert to gather quality information [26]. The first author has experience and professional practice in mental health and intellectual disability as a research instrument. Observational field notes made during the interviews and group discussions captured the behaviour, emotional status, and attitudes of the participants in relation to their experiences of raising the child with ID [24]. All interviews were audio recorded and the credibility of this study was promoted during prolonged interaction with participants in the field between August and January.

### 2.4. Data Analysis

Thematic analysis of data was conducted concurrently with their collection to allow continuous reflection and development of themes in an inductive logical way [22]. The socio-demographic information from both individual and focus group interviews was consolidated and is presented in Table 1 to further explain the characteristics of participants. The audio recorded interviews were transcribed, typed into an Excel sheet, and converted into intelligible statements relevant to both the research problem and the attendant objective of the study [22,23]. Related codes from both focus and individual interviews were allocated to associated categories and uploaded to ATLAS.ti computer programme. A summary was made to indicate of how the two sets of data were connected and complemented each other. Codes were also allocated to the alphanumeric coding list through coding manager assistance. Emerging groups of “families” were allocated similar codes and translated into categories and subcategories [23,25]. The totality of the emergent information from the themes and subthemes was then summarised into meaningful findings that emerged from integration of both individual and focus group interviews as evidence of the study [22]. Additionally, the analysed content of literature and other relevant documents served a complementary function together with the empirically generated evidence such as themes and subthemes in the overall development of the study findings. Guarding against bias of the first author with professional practice and experience in mental health and intellectual disability, the first author’s academic supervisor confirmed emerged themes. Three themes with subthemes emerged on the safety needs of children with ID as experienced by their families.

### 2.5. Ethical Considerations

The study received ethical clearance to commence with the empirical data collection process from the Research Ethics Committee (REC) of the Department of Health Studies at the University of South Africa (reference number, HSHDC/860/2018), formal written permission to collect data at Capricorn District sites from the Limpopo Department of Health (refence number, LP_2018_07_014), as well as the Capricorn Health District (reference number, S.5/3/1/2). All the sampled participants voluntarily signed the researchers’ prepared consent form as an indication of their willingness to participate in the study, and also gave verbal permission for the researchers’ use of the audio recorder during both the in-depth individual interviews and focus group discussion [22].

## 3. Results

The inductive thematic analysis of data revealed a lack of safety of the children with ID. Most families reported a lack of support and poor involvement in the safety of children with ID among members of the nuclear family who were regarded as caregivers, community members, and professionals providing services to these children. The study protected the identity of participants by making use of pseudonyms to present the excerpts.

### 3.1. Theme 1: The Role of Nuclear Family

The study findings revealed that most families raising children with intellectual disabilities were not functioning as a unit to support each family member’s safety. Accordingly, not all family members played active roles towards care and protection of the children with ID. The mothers affirmed poor participation by other family members in ensuring the safety of the children with ID.

#### 3.1.1. Subtheme 1.1: Sibling Interaction

Mothers complained mostly of the behaviour of siblings who were not welcoming of a brother or sister with ID. The following extracts attest to this assertion by the mothers:

“*Only one child does care about her sibling, and she helps me continuously. The other children do not care much, and they separate her from others. If I must go somewhere, I will send her via a taxi to her sister so that so that she can be safe*”.

(Pearl)

“*The other children do not care much. The 11 years old does not understand or like his brother, when asked to give him food, he just throws the plate. I am not satisfied the way he treats him. I am also worried because he is the one who sleeps with him as he sometimes has fits [epileptic seizures] during the middle of the night*”.

(Dinah)

“*The main challenge is in the family; they don’t understand his moods and they say wrong words and sometimes beat him up. They don’t understand him*”.

(Merriam)

Some family members indicated that the safety and security of the children with ID rested upon their shoulders as mothers, more than any other family member. As such, their care burden increased, but they still felt obliged to take the responsibility due to a lack of trust in some of their family members on the safety of their children with ID.

#### 3.1.2. Subtheme 1.2: Fathers’ Role

Mothers reported that fathers were not participating actively as a unit in decision-making regarding the care of their children to ensure safe environments, as attested by the following excepts:

“*It is very difficult. I had conflict with my husband as he once told me to take the child to his biological father. He married me with the child*”.

(Regina)

“*We struggle financially; my husband is not part of the care of the child. My child needs total care, so I must work to support my children as the child is eating a lot*”.

(Pretty)

The results revealed a lack of cohesion in some families where fathers provide less support to their partners to raise their children with ID. There is a lack of fathers’ responsibility to ensure that children’s human needs are met by all family members as primary caregivers.

### 3.2. Theme 2: The Role of the Community

The findings showed a lack of active participation and support from the community towards the safety and protection of children with ID. The families raised a concern in relation to their neighbours and interaction with their peers without ID.

#### 3.2.1. Subtheme 2.1: Neighbour Support Role

Affected families indicated a lack of safety of the children with intellectual disabilities in their own communities who were expected to lend support in raising these children. The families felt that their children were not safe, and were rejected by their neighbours and their peers in the community who did not suffer from any ID. The following statements bear testimony in that regard:

“*Neighbours do not support us. They always tell their children not to play with my child. They thought I would always keep my child in my house. Neighbours are a challenge when it comes to support*”.

(Priscilla)

“*I am forced to be always available to protect the child and make sure that he plays in the yard because if he goes out there is a problem*”.

(Kate)

“*When I ask them why my child is hurt, they [my neighbours] always tell me that they were children playing and I should not be involved as an adult. I need to protect her and make sure she is always happy and safe, as she is my child*”.

(Caroline)

The families expected support from neighbours as part of the community to ensure that their children with ID feel safe around them. The children were rejected by some neighbours who restricted their children’s interaction with children with ID. Some families decided to keep their children behind doors to ensure that they are safe.

The study also found that some community members violated the children’s rights by exploiting and engaging some of the children with ID in physical labour and paid them a pittance, as reflected in the below-cited statement:

“*The child roams around and works for community and they pay him less money. He fetches water for them*”.

(Violet)

The study revealed a lack of understanding of the rights of children with ID by the community members. Some community members took the opportunity of saving expenses by exploiting the children with ID to perform hard labour duties and further taking advantage of their status.

#### 3.2.2. Subtheme 2.2: Interaction with Peers

Families reported that the children were bullied by their peers in the community, taking advantage of their inability to self-protect due to poor insight and judgement:

“*My child sometimes walks around and that is why I worry about his safety. Especially because he cannot fight back when beaten by other children*”.

(Suzan)

“*Some community members do not accept him, and others throw stones at him. I do not understand how they view him. Some parents do not allow their children to play with my child indicating that he is not normal*”.

(Peter)

“*… if they [other children] play with him [my child], he is always crying and when I come out of the house, they run away leaving my child alone. That is hurting me, thus why I keep my child in the house to make sure he is safe*”.

(Mary)

The above-cited excerpts are emblematic of the perception or view that the community tended to marginalise the children with ID, sheerly on the basis of their mental status. Consequently, some of the affected families tended to hide or self-isolate their children from the community as a means to protect them and ensure their safety. Furthermore, these excerpts demonstrate rejection and poor social interactions of these children with their peers without ID.

### 3.3. Theme 3: The Role of Professionals

The affected families reported a lack of concerted professional responsibility in ensuring the safety of their children with ID at school, day care centres, and community health care services.

#### 3.3.1. Subtheme 3.1: Schools and Day Care Centre Facilities

The families referred to negative experiences regarding the safety of their children with ID at schools and day care centres. The following statements attest to their concerns regarding their children’s safety needs that continued to be neglected in the care of professionals:

“*I feel like my child is not safe. After the school transport dropped him, I realised the child was not free until I discovered that it was due to school ill treatment. He was bullied by other learners at school*”.

(Mary)

“*Last time I took him to a certain school, I found that he was scratched on the face and when asked, I was told that they are not with them over the weekend. I once again found his leg swollen and they told me that maybe a bug or some insect bitten him. I was angry because they did not inform me, and I took my child to the clinic*”.

(Agnes)

“*I wish that boys and girls be separated at special schools for the girls to be protected and safe. Nevertheless, I do not trust them because they do not know when they are doing wrong things. Separation will help and the schools will be safe*”.

(Sarah)

Families reported injuries of their children with ID at schools and day care centres. The study revealed a lack of reporting by professionals to their families on the progress of their children with ID. The families were more concerned about the care of these children where the injuries were not prioritised.

Another family member raised further concern about the police services not taking action to investigate reported cases of their child with ID who was sexually abused by another learner with ID at the same school for learners with special needs. This occurrence was articulated thus:

“*I was told by one of the learners that my child was sexually abused by another learner. The teachers did not tell me anything, and that worried me, and I decided to take my child out of the school and reported the matter to the police. When I made follow-up, the case was dropped without explanation. They did not take it seriously*”.

(Maureen)

On the one hand, the above excerpt aptly demonstrates the highly unacceptable and discriminatory behaviour of the police as society’s protectors. On the other hand, the extract above shows that the rights of children with ID, including regarding sexual contact, were not considered to be violated and not taken seriously compared to their peers without ID. The families expected interventions from professionals to ensure that children with ID are protected and feel safe under their care.

#### 3.3.2. Subtheme 3.2: Community Health Care Centres

Families were concerned that the community health providers do not prioritise their children with ID, including nurses and social workers. The children were subjected to waiting in long queues to receive services like any other clients for consultations:

“*With the clinic visits, I have to queue like all patients, and it is difficult to manage the child because he is always running outside*”.

(Kate)

“*The social workers told me the things that I did wrong regarding the care of the child and accused me that I do not take care of my child. I am worried because social worker just judged me without getting part of my story*”.

(Caroline)

This finding gives evidence that health care providers at the clinics were not providing expected support to the families of the children with ID. This revealed a lack of commitment by professionals to prioritise the rights of children with ID to promote their safety.

## 4. Discussion

This study explored the safety needs of children with ID as experienced by family members living with, caring for, and raising these children. Data analysis showed a lack of safety of the children with ID in their home environment, communities, and institutions run by professionals. The families reported the safety needs of their children with ID as those factors that enable their children to always feel safe and secure around them. The social model of disability asserts that a lack of support to the human needs of children with disability impacts on the degree of social inclusivity and their ability to participate actively in their homes and community irrespective of their own bodily incapacity induced by their disability status [6].

A safe home involves support of basic human needs by the family members living in the same households, including fathers, mothers, siblings, and grandparents. A previous study found that in Australia, home was the safest environment for those with ID than any other place [9]. Comparably, the current study findings highlight that home environments were not a safe place for all the children with ID. Some children were subjected to maltreatment by their own family members involved in their care. The study revealed evidence on siblings ill-treating the children suffering from ID whose safety depends on them as family members. This compromised the safe home environment of the children with ID. The findings relate to a Canadian study that found that siblings of children with ID more often presented with mental health disorders, including depression, than those without a sibling with ID [27]. In addition, it is further revealed by a previous study that siblings experience anxiety which results in hostility and anger towards the children with ID [28]. Furthermore, a previous study in the Netherlands found that siblings were more worried about taking over future responsibilities from their parents to care for their sibling with ID [29] which may contribute to maltreatment of their sibling. However, positive attitudes towards a family member with ID requires acceptance of the condition by the whole family [30] as a basis for a safe place to raise them. The study supports that such conversations should be started in the early stages of child development to equip them with knowledge of ID [29].

The study further identified the need for fathers’ roles regarding the safety of children with ID. The findings revealed that mothers undertook the added responsibility of ensuring that the children with ID are safe from internal and external threats or risks. In support of this finding, it was found that in South Africa fathers left their families, avoiding being associated with children with disability [21]. The literature asserts that children with ID have fewer relationships with those nuclear family members who expose them to unsafe home environments [31]. The study highlights less support of mothers by their spouses who were always absent either due to work or failed relationships, leaving all parental responsibilities to mothers. Duran and Ergün [32] found that in Turkey mothers of children with ID were frequently in arguments with their spouses over the care of these children. Most participants were women and either single parents, divorced, widowed, or separated from their spouses, raising the children without support of their partners. Thus, most families in this study were female-headed households, leaving children without father figures. It has been established by research that children with ID from single-parent households suffer a double social stigma and lack of support from their communities [33]. The social stigma exposes children to environments where their safety is not guaranteed. However, this study was conducted in rural areas where most fathers were working far from home, contributing to poor relationships with their children and further weakening the safety of environments of their children with ID. Similarly, in Namibia, spousal support on raising children with ID was a challenge in most families [33]. However, some mothers in this study preferred to stay at home to care for their children with ID on their own to ensure their safety compared to other family members. This increased unemployment in such families, impacting negatively on their financial support.

In African rural environments, children are raised by the community members, making sure that all children feel safe in the presence of neighbours and their peers [19]. However, in this study families lacked support from community members and neighbours regarding the safety needs of the children with ID. Some children with ID were bullied by their typical peers without intervention of the community members. Some neighbours regarded such maltreatment activities of children with ID as a normal part of the child development process. This evidence further indicates a lack of commitment, discrimination, and compromised human dignity of these children from their own communities, affecting their safety needs. In line with this finding, the literature revealed limited contact between people with ID and their neighbours in the Netherlands [34]. Such limited contact exemplifies an implicit exclusion form that effectively bars those with ID from meaningful involvement in neighbourhood activities. Similarly, a previous study found that in Switzerland, some of the children with ID were at risk of being isolated and rejected by their typical peers [35]. However, in Ghana and Zambia, families expected societal inclusion of the children with ID to enhance their safety among their peers and with their neighbours [36]. Provision of safe and nurturing environments to children with ID requires active involvement of communities to prevent abuse and neglect and further protect the children’s citizenship rights crucial for human survival [37].

Most children with ID in rural areas are diagnosed at school, day care centres, and health care facilities where their developmental milestones are compared to their typical peers. However, the findings showed a prevalence of poor professional engagement insofar as the safety needs of the children with ID were concerned at schools, day centres, and health care facilities. The findings revealed that some children were found by their families either injured, victimised, or ill-treated in these facilities under the care of professionals. In line with this finding, a previous study found that children with ID experience rejection in special schools [38]. In this study, victimisation was highlighted by families reporting fewer interventions by professionals to promote safe environments at these facilities. Some families raised sexual violence towards their children with ID at schools which was not reported by the professionals. This finding converged with a previous study that found that school staff members underreported suspicious cases of child victimisation at schools [39] due to a lack of knowledge, attitudes, and communication skills [40]. These findings further resonate with research that found that in South Africa, teenagers with ID were targets of sexual violence [12].

Similarly, a previous study revealed that in the United Kingdom, children with ID were exposed to avoidable and preventable harm under the care of the professionals who did not exercise the same level of care they accorded the children who did not suffer from ID [20]. However, the families took it upon themselves to report the matters to the police who did not pursue the cases. This finding is consistent with those of Greco et al. [39], highlighting that possible victimisation reported to the corresponding authorities for further assessment was withdrawn without interventions. Such a lack of reporting and passive response to such cases provided families with evidence that their children were not safe in these facilities. Regardless of the constitutional rights, educational policies for educators in South Africa are not yet adequately aligned to the protection of the rights of children with ID at schools [13] to make such institutions a safe place. Hence, in this study, some families opted for their children to stay at home, not attending school or day care centres, and risked a bleak future without education for them to be safe, rather than endure ill-treatment and bullying at schools. This further impacted negatively on the children’s rights to basic education. In comparison, a Ghanaian study found that parents preferred to take their children with ID to special schools where they were confident that the safety needs of their children with ID will be fulfilled [41]. The study further revealed that special schools were not exceptionally safe places for children with ID as some children were injured at schools and day care centres. As found by previous studies and this study, further training of educators and support staff on the safety of children with ID and reporting procedures will promote interventions by these facilities to ensure a safe place for these children. Furthermore, implementation of intervention programmes in schools on positive change encourages social interaction between children with ID and their peers without ID [38].

The families reported health care providers who failed to prioritise the safety needs of children with ID. The children were left to roam at the clinics while waiting for consultations during follow up care. The study revealed that most children were not attending health care services as required for monitoring of their development. This was evidenced by use of a snowball technique to trace children with ID during data collection. A previous study found safety inequalities and a lack of advocacy for people with disabilities in the health care systems [42]. Another study indicates that parents of children with ID are expected to manage the medical care and behaviour of their children with disabilities in health care facilities by professionals [43]. The study suggests professional advocacy for children with ID to encourage utilisation of health care services by the children with ID and their families.

However, the results should be interpreted in consideration of the limitations that were noted. Firstly, the research was conducted in underdeveloped rural areas where men generally migrate to other cities in the country for greener pastures. As such, their views and perspectives were not obtained. Secondly, records on the prevalence of children with ID in Limpopo Province were not available which resulted in difficulty in tracing the whereabouts of children with ID and their families. It is in this regard that the snowball sampling method was used to locate families of children with ID who were not known in the health care facilities, schools, and day care centres. Thirdly, the children with ID were not interviewed and their experiences were not voiced. Fourthly, the experiences and knowledge of professionals providing services to children with ID in these institutions were not explored in this study.

## 5. Conclusions

The overall findings in this study prove that that establishment of safe environments in which to raise children with ID is a perennial struggle. The lack of safety and security at home, schools, and day care centres for the children with ID compounds their isolation, rejection, abuse, and exploitation, and brought psychological pain to their families and mothers in particular. Moreover, the limitations induced by their intellectual impairment and inability to protect or fight for their individual rights continuously call for all stakeholders, including their own family members, extended families, neighbours, community members, and professionals, to play critical advocacy roles regarding the safety needs of the children with ID.

## Figures and Tables

**Table 1:** Socio-demographic data of families and the children with ID.

Variable	Category	Number of Participants (*n* = 26)	Percentage (100)
Participants’ relationship to the child	Mother	16	61.5%
	Father	1	3.8%
	Guardian Grandmother Grandfather Aunt Uncle	2 1 1 3 2	7.8% 3.8% 3.8% 11.5% 7.8%
Participants’ children attending schools and day care	Attending	8	30.8%
	Not attending	18	69.2%
Participants’ employment information	Employed	6	23.2%
	Unemployed	16	61.5%
	Pensioner	3	11.5%
	Schooling	1	3.8%
Marital status of participants	Single Married Divorced Separated Widowed	11 7 4 2 2	42% 27% 15% 8% 8%
Participants’ gender	Female	22	84.6%
	Male	4	15.4%

## Data Availability

The data presented in this study are available on request from the corresponding author M.J.M. The data are not publicly available due to information that could compromise the privacy of the research participants.
